# Our Relations to a Few of the Problems in State Medicine1Presidential address at the 36th annual meeting of the Medical Association of Central New York, held at Auburn, September 22, 1903.

**Published:** 1903-11

**Authors:** John L. Heffron

**Affiliations:** Syracuse, N. Y.


### BUFFALO MEDICAL JOURNAL.

VOL. XLIII.—LIX. NOVEMBER, 1903. No. 4.

### ORIGINAL COMMUNICATIONS.

### Our Relations to a Few of the Problems in State Medicine.[1]

BY JOHN L. HEFFRON, A. M., M. D., Syracuse, N. Y.

AT THE opening of this the 36th annual meeting of this association, permit me to thank you for the honor you conferred upon me by making me your president. This association is a voluntary one and has no connection with any other medical body by obligation or by law. It has recognised preëxisting county medical societies and has asked them to select delegates as a matter of compliment, and to secure the most representative medical men in our territory.

There is a great advantage in such an association and we owe much to that wisdom of our founders which determined that this body of medical men should devote itself wholly to scientific improvement, and to the cultivation of a better acquaintance amongst the physicians and surgeons of central New York. Taking up the work in this spirit, your president has the pleasure of saying that, up to this point, the burden of office has been light. In response to the cards mailed in July we are able to lay before you an inviting program of scientific papers.

But now, Ladies and Gentlemen, that I bow my neck to the yoke shaped by our predecessors, I am conscious that it is not an easy one. Presidential addresses to medical societies group themselves usually into two general classes. One deals with the history of medicine and traces the sources of our power; the other brings some fresh contribution of thought to enrich our present possessions. I have chosen neither course, but, at the risk of imposing upon your good nature, I shall raise my voice to a very old tune. The impulse that compels me is the very fact that it is

1. Presidential address at the 36th annual meeting of the Medical Association of Central New York, held at Auburn, September 22, 1903.

such a familiar strain and that there are no new notes to be added. Because we need at times to revive familiar facts and make them burn like the living truths they really are, I shall attempt to discuss

OUR RELATIONS TO A FEW OF THE PROBLEMS OF STATE MEDICINE.

The prevention of disease is the goal toward which the profession is striving. Every solid advance in the definite knowledge of the etiology of diseases and the methods of controlling their causes is a step forward. But technical knowledge alone is not sufficient. It cannot be made effective in a Republic without the support of the people. The instinct for the preservation of one's own life in the presence of immediate or impending danger is uppermost in the breast of every human being, savage or civilised. Humanitarian sentiments that consider the welfare of the race as a whole are rare save in the minds of the most advanced of the civilised nations. In the presence of danger we all revert to the original type. This has been signally illustrated in the history of the great epidemic diseases that have swept our communities. It is to the response from the selfish instinct for self-preservation of the individual life as much as to our perfected knowledge of the methods for controlling the ravages of the deadliest epidemic diseases, that typhus fever and cholera have been swept from the face of the earth and smallpox and yellow-fever have been so greatly controlled.

It is the dramatic quality of events in life which makes the deep and abiding impression. The life that now is in all its fulness and tomorrow is cut off by an epidemic disease so quickly fatal as cholera, is a tragedy that horrifies everyone. No measure, however extreme, will seem too severe to check its deadly onward march. But the demonstration that many other diseases, less acute in their manifestations, and killing not so large a proportion of their victims, and then only after a struggle of weeks or months in weary battle with the resisting forces of the individual, are also infectious and preventable, is the work of the present generation of medical investigators. The pioneers in this field are still living or are scarcely cold in death. It seems incredible that such another period of advance in the etiology and pathology of infectious and communicable diseases can ever again be witnessed in the history of the world as has been recorded in the last half of the XIXth century. Think of it. The cellular pathology of Virchow, that versatile old man but just gone from amongst us, has been established upon a basis of demonstrated fact which seems impregnable. The germ theory of infectious diseases, vaguely foreshadowed since the first epidemic was intelligently observed, and to which Pasteur, only yesterday with us, so nobly contributed, is accepted by the scientific world.

The people, however, have not yet appreciated these facts. So many generations of false teaching, so long a familiarity with the manifestations of typhoid fever, tuberculosis and kindred diseases, so many centuries of the preaching of the damnable doctrine of "The dispensation of a mysterious Providence," have deadened their sensibilities. They witness with apparent equanimity the long procession to a certain grave of thousands of their fellow men annually. They resent every effort to control these plagues which we have demonstrated over and over again are no less real than cholera though less dramatic, if such measures seem to curtail their personal liberty to do as their own lack of knowledge and judgment dictates. They have those amongst them, commonly considered intelligent on other subjects, who through wilful ignorance, I will not say maliciously, cast suspicion on the motives and on the worth of scientific medical investigators, and prejudice the public mind against their teachings and their reiterated warnings. They have journals, so attractive in their legitimate fields as to secure the widest circulation, that apparently delight in periodic attempts at ridicule of such immortals as Jenner, Virchow, Pasteur and others whose work we are proud to recognise as of the greatest value to humanity. It is against such blind and wilful ignorance, such malicious minds, such prejudice and much lawlessness that the state department of public health, of which we are coadjutors, is compelled to array itself in an attempt to carry forward the work of preventing and controlling the ravages of typhoid fever and tuberculosis and of keeping from the people a renewal of the horrors of epidemic smallpox.

TYPHOID FEVER.

Our country enjoys the unquestioned distinction of having contributed more to the definite knowledge of typhoid fever than any other nation in the world. The identity of the disease, the nature of the infectious germ, its various modes of distribution, the laws of its growth and multiplication, its distribution in an infected individual, its usual and unusual seats of activity, the effects of its products, and the ways of preventing its entrance into the human body, are definitely known. There is no longer any mystery about it. The demonstration is as complete as is that of any problem in mathematics. Other nations have taken intelligent action upon this solid foundation of knowledge and the empires and kingdoms of the continent of Europe have greatly diminished its autumnal sacrifices. We, with a form of government which derives its power from the people, are a whole generation behind them. Our annual visitations of typhoid go on apparently unheeded. The strength and glory of our young men are ruthlessly cut down with little thought that it is an unnecessary sacrifice. Our beautiful lakes, majestic rivers, and babbling brooks are rendered deadly; and not until some widespread epidemic of the disease in a prominent community occurs, do we consider deeply the awfulness of this condition of things.

Since our last meeting what an appalling illustration of this fact has this section of our state in particular, and the entire country in general, had in the epidemic of typhoid at Ithaca. While some in many communities are questioning the accuracy of the assertions of scientists, and others are hesitating to impose upon their city or village the seemingly heavy burden of expense necessary to prevent the occurrence of such an epidemic, it will pay us to stop a moment and consider some phases of this epidemic. No official report of it has yet been attempted. Because the population of Ithaca includes large numbers of university students, many of whom left the city in the early days of the epidemic, it will be difficult to collect accurate statistics. From facts published in the newspapers and in the reports of the president of Cornell University and from direct information, it would seem that there were at least 1,400 cases. The reported deaths among the students was 10 per cent. of those who were known to have the disease. No accurate figures for the total deaths from the epidemic can be obtained, but it is fair to assume that the rate was not less than that exhibited among the students of Cornell.

The epidemic was directly traced to an infected water supply owned and operated by a private company. The impounded water was supplied by creeks running through farm lands, and the watershed was not under any supervision by the company. Upon investigation this watershed was found to present every possible form of pollution to the stream which drained it, though the specific infection is supposed to have come from some of the workmen engaged in the construction of a new dam. The epidemic greatly crippled the work in Cornell University. It paralysed the business of Ithaca and entailed unmeasurable anxiety upon all her citizens. The story of the suffering and grief which it caused could not be told in a book. If accurate figures were at hand it would be possible to show what was the entire cost in money of this epidemic. Such an argument is of value because so many think only or largely of the money that is required to secure needed sanitary measures for a city. A half million dollars would seem an enormous sum to such, if a proposition to appropriate that amount were made to secure a wholesome supply of water for a city of the size of Ithaca; but I will venture to predict that when some future statistician shall gather together the items of the expense of this epidemic to individuals, to the university, to the city and to the state, that sum will be greatly exceeded. This same lesson has been taught in the same heartbreaking way many times before and it will doubtless be repeated, with all its attending horrors, many times to come, but, if we in central New York shall have learned it this once for all time, this epidemic will prove to us a blessing.

There are certain measures which we must demand from the state or from such governmental bodies as control watersheds, if we are to have a pure water supply. To prevent typhoid fever the state must first compel the protection from pollution of all kinds of all watersheds which drain into waters granted to municipalities for public water supplies, and it must compel all private corporations, to which it grants permission to furnish water to a community, to comply with this provision. It must compel the sterilisation of all sewage before it is discharged into public waterways that are sources of water supply. Think of the present condition of the majestic St. Lawrence, the picturesque Hudson, and the beautiful Black River, and the charming lakes of our own vicinity and of the Adirondacks! The people have a right to unpolluted lakes and rivers and streams and the time has come when they should demand them. Because practically all of our available sources of water supply are already infected, it must compel the instalment of competent purification plants as adjuncts to all water systems. It must compel the abandonment of all wells in cities or villages furnished with wholesome water.

Our individual responsibilities are not slight or valueless. In every case of typhoid we must seek earnestly to trace the source of infection and we must not rest content with negative evidence. Remember the brilliant demonstration of Dr. Hutchings, of the Ogdensburg State Hospital. After many failures to locate the source of infection in an epidemic in that hospital a year ago, he finally traced it to infected ice and cultivated the bacilli from ice that had been stored nine months. We must instruct our nurses in the methods which will absolutely sterilise the various excreta before they are disposed of in any way whatsoever, and we must not take it for granted that they are masters of such methods, from whatsoever training school they may have received their diplomas. We must see that the clothing used on or about the patient is properly disinfected before going to the laundry. We must know that everything which is consumed by the patient and his family is so prepared that, if infected, it will be rendered sterile. We must second the efforts of our local and state health officers and stimulate and encourage their efforts to safeguard the health of the people. By such individual and combined action we can hasten the day when the people will move with resistless strength against this enemy, which is so vulnerable and so exposed.

TUBERCULOSIS.

The problem of the control of tuberculosis is now generally conceded to be one of the important problems of state medicine. Your president has so recently reviewed this subject that he will spare you a reiteration of facts so well known to you all. Since last January some notable contributions to the prognosis of tuberculosis have been made which deserve particular mention and emphasis. Hitherto the opinion has been that, while an individual might recover from the disease, he was insecure in his tenure of life and in almost constant danger, certainly in greater danger than the healthy man, of a recurrence of the disease. In a notable paper at the Congress of American Physicians held at Washington last May, Trudeau called attention to the fact that his infected animals in which he had obtained a cure, were apparently immune to a reinfection of tuberculosis. In a private letter on the subject of immunity to the writer he said, "a really healed lesion may increase the resistance or immunity, as few of the 60 per cent. who have old lesions seem to relapse." Maragliano, in his paper before the World's Congress of Physicians at Madrid last spring, said, "when a patient is surely and completely cured of a pulmonary tuberculous focus, with the formation of a sclerotic tract in the area of a previous infection, he generally remains thereafter permanently unsusceptible to tuberculosis." If this conclusion shall prove to be true, what a new aspect of hopefulness and what added impulse to our enthusiasm in the combat against this disease will be conferred upon us! But the accent in all communications is on the "really healed." It will require our keenest powers and most accurate methods to determine this point. An eminent physician who has a tuberculous focus, quiescent for many years, accidentally inhaled some tuberculin he was making. He developed a most marked and distressing reaction which incapacitated him from work for some time. This incident illustrates not only the fact that a quiescent focus is not of necessity a healed lesion but also that in a supposedly healed lesion, when other methods of discrimination fail, the truth may be elicited by the employment of Koch's original tuberculin.

The fear that came upon us lest Koch's latest word concerning the nonintercommunicability of human and bovine tuberculosis and his illogical conclusions from his observations, might call a halt in the crusade against tuberculous cattle, was happily unfounded. The last word has not yet been said on that subject, but enough facts have been accumulated to sustain health boards in their work. Independently of the question of the intercommunicability of this disease, the people insist upon protection against the products of cattle suffering from disease of any kind whatsoever, and so there is little danger that the work of exterminating tuberculosis amongst dairy herds will cease until that object has been accomplished.

In the control of tuberculosis the time has come when we need more activity on the part of the physicians in general. The various state sanatoria have proven entirely successful from whatever point they may be viewed. Our own state sanatorium will prove an inestimable benefit even though it can accommodate so few of our infected poor, and will mark the beginning of the proper care of these unfortunate ones. The duty of the state is to so care for the tuberculous who are a public charge that they can have every advantage for the curative treatment of this disease. Such a state of things cannot be brought about by establishing special hospitals in connection with institutions for the housing and care of the indigent and helpless poor. There must be in every city and in the center of every territory with a given population, a special hospital for tuberculosis to which patients will be attracted by the superior advantages for recovery and to which it will be considered no stigma to be taken.

Our personal duty in hastening a solution of this problem is, first of all, to educate every sense and cultivate every method by which an early diagnosis can be established, for the prognosis is proportionally more favorable the earlier the diagnosis is made, and he is not fit to practise medicine who cannot diagnosticate tuberculosis until the bacilli are demonstrated to be present in the sputum. Having established in ourselves an enthusiastic belief in the curability of tuberculosis we are prepared to communicate it to our patients and to the public, and to teach them every detail of personal hygiene and public precaution necessary to be carried out. The records of the many institutions devoted to the care of the tuberculous are easily accessible to every physician. Armed with these facts, which prove the curability of the disease and the great financial gain to the public from such cure, we are prepared to go forward and hasten the day when the tuberculous poor of every community shall be properly treated.

SMALLPOX.

The dangers of the recurrence of epidemic smallpox are unquestionably increasing. The old adage says, "the constant dripping of water wears away the stone." The public utterances of those who oppose vaccination, now more brazen than for half a century, and the sense of security resulting from nearly a hundred years of deliverance from this awful pest, have not been without an unwholesome influence. The rigidity with which our laws governing vaccination have been enforced has been relaxed and our sense of personal responsibility has perceptibly diminished. How many family physicians now vaccinate the infants who enter the world by their help? And yet that was considered an obligation a generation ago. In how many communities where vaccination is required before a child can enter the public schools is equally strict attention paid to the children who enter parochial or private schools? The law requiring revaccination after the establishment of puberty is essential if a community is to be really protected, but is that law carried out? In how many institutions for higher learning and in how many factories and stores employing large numbers of people is any such qualification as recent and successful vaccination required?

Our relations to this problem may be briefly summarised. We must not be influenced by the ignorant clatter of the antivaccinationists. We must be ready "with chapter and verse" to show the wavering that immunity against smallpox is conferred by vaccination. The arguments against vaccination today are no different and no more potent than those which assailed Jenner and over which he so signally triumphed. It is an inspiration to re-read the records of his life, to bring before our imagination the picture of those devastating years before his immortal discovery, and to contrast with them the records of the nearly completed century since vaccination was carried out in all christendom. We would commend to all opponents of vaccination (and to all friends too for that matter) the perusal with an open mind of that number of the *British Medical Journal,* published July 5, 1902, and we would ask *Life* to republish for the benefit of its many readers the graphic illustrations of the death-rate before and since the advent of vaccination which appeared in the article of Arthur Newsholme on "The Epidemiology of smallpox in the XIXth century." The abuse of vaccination by careless operators can and should be checked. The simple laws of surgical asepsis are as necessary here as in major operations. The operation of vaccination should be so carried out that no secondary infection can be possible. We must insist on surgical cleanliness in the operation and that we be supplied with a virus free from all impurities, yet not emasculated. It is my opinion that such virus should be prepared and distributed under the direct supervision of a state officer and in no other way. We must see that the children in the families we attend are vaccinated early in life and again after the establishment of puberty, and we must support the local boards of health in the performance of their whole duty in this important matter.

All this is trite. It has been said over and over again. It will be repeated many times to come. The hearts of communities will bleed again and again because of their punishment for their sins against God's laws of sanitary living. But there shall come a time,—how far off we cannot foresee,—when the eyes of the people shall be opened, their ears unstopped, and their hands shall eagerly work out their own physical salvation.

528 S. Salina Street.

